# Author's response—I

**Published:** 2006

**Authors:** Shridhar Sharma

**Affiliations:** * Professor Emeritus National Academy of Medical Sciences and Institute of Human Behaviour and Allied Sciences, Delhi; e-mail: sharma.shridhar@gmail.com

Sir,

Dr Debashis Chatterjee raises four points, regarding my commentary on Amit Ranjan Basu's article on ‘Historicizing Indian Psychiatry’,^1^ while doing so, he misses some of the basic issues. I would briefly reply the same:

1. One of the basic observation in my article was that the early establishment of mental hospitals in the Indian subcontinent reflected^2^ the need and demand of ‘European patients’ and to manage and treat Englishmen and Indian Sepoyees employed by the British East India Company. The location of the first hospitals, were in proximity, where the battles were fought.
2. Secondly, his view that ‘Indian civilization and *Ayurveda’* is not antedated but ‘post-Alexander’ and is a product of Greek civilization is possibly based on the source of his information and probably based on colonialistic viewpoint. The study of history is not simply a restoration of the past events but is also its proper evaluation in a most objective manner. This is a challenging task, as there is no authentic source book of ancient Indian civilization. We greatly depend on the observations written by western writers, guided by different approaches, viz. (a) the orientalist, viewpoint, (b) the administrative perceptions, and (c) the missionary writers approach, and also (d) by communist writers' perception. Any objective reconstruction of the history of civilization may take different paths and different conclusions. This viewpoint is beautifully illustrated by the two radically different forms taken by two group of writers are: one, the *History of mankind* published by UNESCO and second the series published by the Columbia University Press under the title *Introduction to Oriental Civilizations*.
  The *Ayurveda* is believed to be *upveda* or a branch of *Atharvaveda*, in this we find ample references to the medicine, principles of treatment and descriptions of the different parts and organs of the human body, and the time is calculated as one to two thousand years before Christ. The exact time of *Atreya* and *Sushruta* who contributed richly to Ayurveda can not be definitely fixed in the absence of necessary evidence but there is a general inference that *Atreya* belonged to the 6th or 7th century BC. These periods are agreed to, as there is material available for fixing the time of Lord Buddha, who was born in 557 BC and in the literature of that time there is reference to two great Indian universities one at *Kasi* and another in *Takshashila* where *Ayurveda* was taught. This helps us to fix the time of *Ayurveda*.
3. Thirdly, there is rich evidence regarding the multitude of psychotherapeutic theories and practices, in vogue in ancient India. Due to paucity of space, I would mention only about *Mantra vidya. Mantra vidya* is a major, self-sufficient and independent method of Indian psychotherapy and it is one of the oldest methods. *Mantra* is related to *manas* or mind. *Mantra* may be in the form of coherent words or single letters, arranged in a certain order. It is not only a conjoined word but a particular sound body of consciousness with a definite meaning and energy in itself. According to some, it is the sound body, a power charged with the intense vibrations of the spiritual personality of the creator or the seer of the mantra. This therapy is in usage since *vedic* times and is a valuable tool, which is practiced widely in India.
  *Mantra* are of two types
  - *Vedic mantra* and
  - Tantric mantra
  The *vedic mantras* are older and derived from *vedas* are longer and have a meaning while *tantric mantras* are of later origin and are used for *japa* (repetition) and for keeping with oneself in the form of *yantra*.
4. Similarly, various techniques of ‘suggestion’ were used in ancient India, when mesmerism was popular in Europe, it was an Indian priest Abba de Faria, a Hindu Brahmin from Goa, who later converted to Christianity and challenged Mesmer and explained the mechanism of ‘mesmerism’ on the doctrine of suggestion. Dr Egas Moniz, a nobel laureate has written a biography on Abba de Faria. Much later, an English doctor James Esdale, who operated with the help of hypnosis in one of the hospitals in Calcutta. Similarly the practice of introspection and autosuggestion techniques were in practice. It is well documented that Buddha did various meditative practices and underwent ascetic austeries and practiced the highest contemplation before giving his first sermons in Sarnath. The practice of introspection and autosuggestion techniques are still followed in Japan, such as Nakian therapy,^3^ a type of psychotherapy based on introspection and self-reflection or Buddhism Principles.
5. The philosophy of yoga, propounded by *Patanjali* 500 BC, clearly mentions about the spiritual component and selfrealization and individual salvation. The concept of spirituality is certainly not a post-colonial construct.

## References

[1] Sharma S (2006). Psychiatry, colonialism and Indian civilization: A historical appraisal. Indian J Psychiatry.

[2] Basu AR (2005). Historicizing Indian Psychiatry [Viewpoint]. Indian J Psychiatry.

[3] Nakamuri Kei (2006). The History of Psychotherapy in Japan. Int Medical Journal.

